# Macro-adénome surrénalien masquant une hyperplasie micronodulaire des surrénales au cours d’un syndrome de Cushing ACTH indépendant et hypokaliémie réfractaire

**DOI:** 10.11604/pamj.2017.26.230.12053

**Published:** 2017-04-25

**Authors:** Wafa Alaya, Haifa Bouchahda, Asma Fradi, Baha Zantour, Mohamed Habib Sfar

**Affiliations:** 1 Service d’Endocrinologie et Médecine Interne, Hôpital Tahar Sfar, Mahdia, Tunisie

**Keywords:** Syndrome de Cushing, hyperplasie micronodulaire des surrénales, adénome corticosurrénalien, hypokaliémie, Cushing’s syndrome, micronodular adrenal hyperplasia, adrenocortical adenoma, hypokalemia

## Abstract

L’association d’une hyperplasie micronodulaire des surrénales à un macro-adénome surrénalien au cours d’un syndrome de Cushing (SC) ACTH-indépendant est rare et suscite beaucoup de questions. Nous en rapportons un cas. Patiente âgée de 35 ans nous a été adressée pour suspicion d’un SC devant une obésité facio-tronculaire avec hypokaliémie d’origine rénale. Les explorations hormonales ont objectivé un SC ACTH-indépendant et le scanner abdominal a montré macro-adénome surrénalien gauche de 2cm avec une surrénale droite normale. La patiente a eu une surrénalectomie gauche. Cependant, l’hypercortisolisme et l’hypokaliémie ont persisté. L’examen anatomopathologique a permis de conclure à un adénome corticosurrénalien de 2,5cm, avec une hyperplasie micronodulaire non pigmentée des surrénales (i-MAD). La patiente a eu une surrénalectomie droite, suivie d’une insuffisance surrénalienne. Paradoxalement, l’hypokaliémie a persisté sans autres anomalies ni explication évidente (magnésémie, pH sanguin et urinaire, bilan phosphocalcique et échographie rénale normaux) nécessitant une supplémentation parentérale puis orale par du KCl.

The association between micronodular adrenal hyperplasia and macro-adrenal adenoma in patients with ACTH-independent Cushing’s syndrome (CS) is rare and raises a lot of questions. We here report the case of a 35-year old female patient referred to us for suspected CS due to central obesity associated with renal hypokalaemia. Hormonal explorations objectified ACTH-independent CS and abdominal CT scan showed left macro-adrenal adenoma measuring 2cm in diameter associated with normal right adrenal gland. The patient underwent left adrenalectomy. However, hypercortisolism and hypokalaemia persisted. Anatomo-pathological examination allowed the diagnisis of adrenocortical adenoma measuring 2,5cm in diameter associated with unpigmented micronodular adrenal hyperplasia (i-Mad). The patient underwent right adrenalectomy followed by adrenal insufficiency. Paradoxically, the hypokalaemia persisted without other abnormalities nor obvious explanation (normal magnesium, urine pH, blood pH, phosphocalcic assessment and renal ultrasound) requiring parenteral and oral KCl supplementation.

## Introduction

Les hyperplasies ou dysplasies micronodulaires bilatérales des surrénales constituent une cause rare du syndrome de Cushing (SC) (moins de 2% des cas). L’association d’une hyperplasie micronodulaire des surrénales à un macro-adénome surrénalien au cours d’un SC ACTH indépendant n’est pas fréquente et suscite beaucoup de questions. Nous en rapportons un cas qui était en plus particulier par la persistance d’une hypokaliémie après surrénalectomie bilatérale.

## Patient et observation

Patiente âgée de 35 ans, asthmatique sous ventoline, a été adressée à notre service d’Endocrinologie pour suspicion d’un SC devant une obésité facio-tronculaire et une hypokaliémie d’origine rénale (kaliémie= 2,9 mmol/l, kaliurèse= 40 mmol/24h) révélée par des paresthésies des extrémités avec asthénie. Des dosages de la cortisolémie de base demandés par un médecin en ville, ont montré des valeurs élevées: 890ng/ml et 383ng/ml. La patiente nous a alors été adressée pour complément d’exploration et prise en charge. A l’examen physique, la patiente avait un indice de masse corporelle à 38kg/m^2^, un tour de taille à 87 cm et une bosse de bison. Elle n’avait pas de signes d’hypercatabolisme protidique cutané, ni d’hirsutisme. Sa pression artérielle était à 12/7 cm Hg, contrôlée à plusieurs reprises, et le reste de l’examen était sans particularités. Les explorations hormonales ont montré une inversion du rythme nycthéméral du cortisol (Cortisolémie à 8h= 180 µg/l, Cortisolémie à 20h= 273 µg/l), avec absence de freination de la sécrétion du cortisol au freinage minute (cortisolémie après freinage minute= 314µg/l). Le cortisol libre urinaire était supérieur à 4 fois la normale (421 µg/24h) et l’ACTH était inférieure à 5 ng/l, permettant de retenir le diagnostic d’un SC ACTH-indépendant. Le scanner abdominal a montré une formation arrondie, hypodense, bien limitée, de 2 cm de diamètre écartant les deux bras de la surrénale gauche. La surrénale droite avait un aspect tomodensitométrique normal. Un SC ACTH-indépendant secondaire à un adénome surrénalien a été retenu. La patiente a eu une surrénalectomie gauche. Cependant, en postopératoire, la cortisolémie est restée élevée (204,7 µg/l), non freinable sous freinage minute (152,5 µg/l), avec persistance de l’hypokaliémie. L’examen anatomopathologique de la pièce opératoire a conclut à un adénome corticosurrénalien de 2,5 cm de grand axe, avec présence de plusieurs autres nodules de même aspect histologique et de taille infra-centimétrique au sein du reste du parenchyme surrénalien. L’exploration du système rénine angiotensine aldostérone était sans anomalies (en position couchée: aldostéronémie= 14pg/ml et réninémie= 20 mU/l; en position debout: aldostéronémie= 269 pg/ml, réninémie= 98,6mU/l, rapport aldostéronémie/réninémie= 4). Le diagnostic d’une hyperplasie micronodulaire non pigmentée des surrénales (i-MAD), non détectée par la TDM abdominale a été retenu. La patiente a eu une surrénalectomie droite qui était suivie d’une insuffisance surrénalienne (cortisolémie de base en postopératoire= 17 µg/l). L’examen anatomopathologique de la deuxième pièce opératoire a confirmé l’i-MAD, en montrant au sein du parenchyme surrénalien plusieurs nodules bien limités non encapsulés dont la taille varie de 1 à 3 mm, formés par des cellules claires similaires à celles du cortex surrénalien et ayant une architecture fasciculée ou glomérulée. Le reste du tissu surrénalien était sans lésions histologiques notables. Paradoxalement, l’hypokaliémie a persisté en postopératoire. Elle était sévère (< 2,5 mmol/l), associée à une hyperkaliurèse inappropriée (>25 mmol/24h) et ayant nécessité une correction par voie intraveineuse. Les autres explorations biologiques ont montré un pH sanguin, une calcémie, une phosphorémie et une magnésémie normaux avec une calciurie à 1,5 mmol/24h et un pH urinaire à 6. L’échographie rénale était aussi sans anomalies, particulièrement sans néphrocalcinose. Au bout d’une dizaine de jours le relai par voie orale de la supplémentation potassique a été réalisé à la dose de 2g/jour de KCl, permettant le maintien d’une kaliémie normale.

## Discussion

Notre observation nous expose à deux problématiques: d’une part l’association d’un adénome surrénalien à une i-MAD au cours d’un SC ACTH-indépendant, et d’autre part l’hypokaliémie persistante après la surrénalectomie bilatérale. L’association d’un adénome surrénalien à une i-MAD chez notre patiente soulève plusieurs questions: l’adénome cortico-surrénalien était-il fonctionnel ? S’il était fonctionnel, est ce qu’il y a une explication physiopathologique à cette association et quelle est sa fréquence? Et est ce qu’il était possible de suspecter l’i-MAD avant la première surrénalectomie? Le caractère fonctionnel de l’adénome surrénalien aurait pu être élucidé par une scintigraphie à l’iodocholestérol ou par une étude immuno-histochimique de l’adénome, testant l’immuno-réactivité de la 17a hydroxylase, la 21 hydroxylase et l'ARNm de la CYP1β. Cependant, la scintigraphie n’a pas été réalisée car la question ne s’est pas posée initialement et les données cliniques et para-cliniques initiales étaient en faveur d’un SC ACTH-indépendant lié à un nodule surrénalien. L’immuno-histochimie n’a pas été réalisée car nous ne disposons pas des anticorps nécessaires pour cet examen. Après la surrénalectomie gauche, l’intensité biologique de l’hypercorticisme a nettement diminué (cycle nycthéméral du cortisol devenu respecté et diminution de la cortisolémie après freinage minute). Cette diminution pourrait être en rapport avec la surrénalectomie et pas nécessairement avec l’adénomectomie. Enfin l’étude anatomo-pathologique de la surrénale gauche a montré un aspect histologique identique de l’adénome surrénalien et des autres micronodules, ce qui laisse supposer que les micronodules et le macroadénome avaient la même fonctionnalité. Concernant la fréquence de l’association d’un adénome surrénalien à une i-MAD, nous n’avons pas trouvé dans la littérature de publication précisant cette fréquence ni d’autres observations la rapportant. Un seul cas publié à rapporté l’association d’un nodule surrénalien ectopique à une hyperplasie micronodulaire des surrénales, au cours d’un SC ACTH-indépendant [1]. Selon Stratakis CA, qui a particulièrement publié sur la pathologie surrénalienne au cours du SC ACTH-indépendant, des macro-adénomes surrénaliens uniques peuvent être rencontrés au cours de l’hyperplasie micronodulaire des surrénales. Cependant, selon le même auteur, la plupart des patients sont soit macro soit micro-nodulaire et il existe rarement un continuum entre ces deux types d’atteinte chez le même sujet [2]. En effet, bien que toutes les formes d’hyperplasie bilatérale des surrénales étudiées jusqu'à présent (miconodulaires pigmentées ou non et macronodulaires) semblent être liées à l'augmentation de la signalisation de l'AMPc, les modifications histopathologiques et fonctionnelles des surrénales varient considérablement selon le type de mutation qui a déterminé cette augmentation [2-6]. Il s’en suit que chez notre patiente on peut émettre l’hypothèse que l’association de l’adénome surrénalien à l’hyperplasie micronodulaire des surrénales résulte de deux types de mutations différents.

Concernant la possibilité de suspicion diagnostique de l’i-MAD avant la première surrénalectomie, l’hyperplasie micronodulaire bilatérale des surrénales peut passer complètement inaperçue à la TDM quand les micronodules sont de taille inférieure à 3mm [7] et c’était le cas de notre patiente. En plus, jusqu’à nos jours il n’a pas été rapporté de particularités hormonales évocatrices de l’i-MAD. Ceci en comparaison avec l’hyperplasie micronodulaire pigmentée des surrénales où l’augmentation paradoxale de la cortisolémie au décours du test de Liddle constitue un outil diagnostique [2]. La découverte en 2^ème^ lieu de l’hyperplasie micronodulaire des surrénales chez notre patiente, avait causé la fragmentation du traitement chirurgical en deux temps. Ceci soulève la question de l’intérêt d’indiquer systématiquement la scintigraphie à l’iodocholestérol en cas de SC ACTH-indépendant. Par ailleurs, pour le problème de l’hypokaliémie d’origine rénale, persistante après la surrénalectomie bilatérale, l’origine médicamenteuse à été éliminée par l’interrogatoire. Les causes endocriniennes liées à un reliquat tumoral ou par sécrétion ectopique de cortisol ou d’aldostérone ont été écartées puisque la cortisolémie était effondrée après la 2^ème^ surrénalectomie et l’exploration du système rénine angiotensine aldostérone était normale. Une hypokaliémie par néphropathie tubulaire a été alors recherchée, liée à une hypomagnésémie, à un syndrome de Bartter, à un syndrome de Gitelman ou à une acidose tubulaire. Cependant la magnésémie était normale et les autres diagnostics évoqués étaient peu probables vue l’absence de manifestations cliniques en faveur (surdité et retard de croissance pour le syndrome de Bartter et l’acidose tubulaire) et vue la normalité du pH plasmatique, l’absence d’hypercalciurie et de néphrocalcinose (ces deux dernières manifestations étant rencontrées au cours du syndrome de Bartter et de l’acidose tubulaire). Deux cas d’hypokaliémie persistante après surrénalectomie bilatérale pour SC ACTH-dépendant paranéoplasique ont été rapportés dans la littérature [8, 9]. Les auteurs de ces observations ont retenu comme cause de cette hypokaliémie un déficit de l’activité de la 11βhydroxystéroide déshydrogénase qui normalement inactive le cortisol en cortisone. Ceci entraine une accumulation du cortisol, y compris celui apporté par l’hydrocortisone donnée pour la substitution de l’insuffisance surrénalienne postopératoire, avec une exagération de son effet minéralo-corticoïde. Cette hypothèse a été attestée par la disparition de l’hypokaliémie après le remplacement de l’hydrocortisone par la dexaméthasone dans les deux cas rapportés. La dexaméthasone n’a pas été testée chez notre patiente car ce médicament n’est pas commercialisé dans notre pays.

## Conclusion

La visualisation d’un adénome surrénalien au cours d’un SC ACTH-indépendant n’élimine pas l’hyperplasie micronodulaire des surrénales. La réalisation systématique d’une scintigraphie à l’iodocholestérol aurait pu permettre une découverte plus précoce de l’i-MAD et éviter la fragmentation du traitement chirurgical chez notre patiente. L’hypokaliémie réfractaire après la surrénalectomie bilatérale reste mal expliquée.

## References

[1] Louiset E, Gobet F, Libe’ R, Horvath A, Renouf S, Cariou J (2010). ACTH-Independent Cushing’s Syndrome with Bilateral Micronodular Adrenal Hyperplasia and Ectopic Adrenocortical Adenoma. J Clin Endocrinol Metab.

[2] Stratakis CA (2008). Cushing syndrome caused by adrenocortical tumors and hyperplasias (corticotrophin-independent Cushing syndrome). Endocr Dev.

[3] Stratakis CA, Boikos SA (2007). Genetics of adrenal tumors associated with Cushing’s syndrome: a new classification for bilateral adrenocortical hyperplasias. Nat Clin Pract Endocrinol Metab.

[4] Fragoso MC, Domenice S, Latronico AC, Martin RM, Pereira MA, Zerbini MC (2003). Cushing’s syndrome secondary to adrenocorticotropin-independent macronodular adrenocortical hyperplasia due to activating mutations of GNAS1 gene. J Clin Endocrinol Metab.

[5] Bertherat J, Groussin L, Sandrini F, Matyakhina L, Bei T, Stergiopoulos S (2003). Molecular and functional analysis of PRKAR1A and its locus (17q22–24) in sporadic adrenocortical tumors: 17q losses, somatic mutations, and protein kinase A expression and activity. Cancer Res.

[6] Horvath A, Giatzakis C, Robinson-White A, Boikos S, Levine E, Griffin K (2006). Adrenal hyperplasia and adenomas are associated with inhibition of phosphodiesterase 11A in carriers of PDE11A sequence variants that are frequent in the population. Cancer Res.

[7] Rockall AG, Babar SA, Aslam Sohaib SA, Isidori AM, Diaz-Cano S, Monson JP (2004). CT and MR Imaging of the Adrenal Glands in ACTH-independent Cushing Syndrome. RG.

[8] Arteaga E, Fardella C, Campusano C, Cárdenas I, Martinez P (1999). Persistent hypokalemia after successful adrenalectomy in a patient with Cushing's syndrome due to ectopic ACTH secretion: possible role of 11beta-hydroxysteroid dehydrogenase inhibition. J Endocrinol Invest.

[9] Campusano C, Arteaga E, Fardella C, Cárdenas I, Martínez P (1999). Cushing syndrome by ectopic ACTH secretion: analysis of the physiopathologic mechanism of hypokalemia. Report of two cases. Rev Med Chil.

